# PD150 How Is Genetic Testing Evaluated? An Updated Systematic Review Of Assessment Frameworks

**DOI:** 10.1017/S0266462324003830

**Published:** 2025-01-07

**Authors:** Valentina Baccolini, Giuseppe Migliara, Antonio Sciurti, Erica Pitini, Anna Ewa Kaminska, Valentina Soccodato, Jessica Iera, Immacolata Leone, Carolina Marzuillo, Paolo Villari

## Abstract

**Introduction:**

Assessment of the risks and benefits of genetic and genomic tests has long been addressed using ad hoc evaluation methods. They are mostly ACCE-based, focus on technical aspects, and often overlook economic and organizational considerations. The few health technology assessment (HTA) based approaches, though more comprehensive, lack validation and implementation. This review’s purpose was to identify evaluation frameworks for genetic and genomic tests and to synthesize their key aspects.

**Methods:**

PubMed, Scopus, Web of Science, Google Scholar, and Google Search were used to identify records describing any assessment framework for genetic or genomic tests. As this was an update of a previous systematic review, the search was restricted to records published from 1 October 2020. Inclusion criteria were documents describing evaluation frameworks for genetic or genomic tests that were original, specifically created, and covered at least three evaluation components (analytic validity, clinical validity, clinical utility, economic aspects, or ethical, legal, and social implications). This study was supported by the European Commission and the Ministry for Universities and Research under the National Recovery and Resilience Plan (M4C2-I1.3 Project PE_00000019 “HEAL ITALIA”).

**Results:**

Overall, 22,862 records were retrieved and 12,546 unique records were screened, of which 67 documents were assessed for eligibility. However, none of these met the inclusion criteria and no additional framework was found. In contrast, a total of 37 studies reporting 30 different frameworks were included from the previous systematic review. The analysis of these frameworks revealed that they were published between 2000 and 2019 and were mostly based on the ACCE model (n=13), on the HTA process (n=6), or both (n=3). Others referred to the Wilson and Jungner screening criteria (n=3) or to a mixture of different criteria (n=5).

**Conclusions:**

A pressing need exists for a universally accepted evaluation framework for genetic and genomic tests. A shift from ad hoc assessments to a general HTA methodology, potentially based on the EUnetHTA Core Model®, is needed. By integrating solid theoretical and methodological principles, a validated, comprehensive, and widely shared tool for evaluating genetic tests can be realized, promoting consistency across Europe and beyond.

